# Impact of post-admission changes in potentially inappropriate medication use on risk of subsequent hospitalization among nursing home residents

**DOI:** 10.3389/fphar.2025.1655681

**Published:** 2025-09-23

**Authors:** Jing Zhao, Hyunwoo Chae, Young-Mi Ah, Kwanghee Jun, Ju-Yeun Lee

**Affiliations:** ^1^ College of Pharmacy and Research Institute of Pharmaceutical Sciences, Seoul National University, Seoul, Republic of Korea; ^2^ College of Pharmacy, Yeungnam University, Gyeongsan, Gyeongbuk, Republic of Korea; ^3^ College of Pharmacy, Research Institute of Pharmaceutical Science, Gyeongsang National University, Jinju, Republic of Korea

**Keywords:** potentially inappropriate medication, utilization pattern, hospitalization, nursing home, claims data

## Abstract

**Objectives:**

As the population of nursing home (NH) residents grows, the management of polypharmacy and potentially inappropriate medications (PIMs) becomes crucial. Limited research exists on how changes in PIM use affect adverse outcomes, or the benefits of reducing such medications. This study explores post-admission trajectory of PIM utilization and polypharmacy, and their association with hospitalization risk.

**Methods:**

Analyzing national claims data from 23,982 seniors aged ≥65 admitted to NHs from 2008 to 2018, we assessed PIM utilization based on the 2019 Beers criteria and calculated the total number of medications prescribed during the first month of each quarter in the year following NH admission. We then used a cause-specific hazard model to explore how changes in the number of medications and PIM use were associated with the risk of hospitalization in the following 2 months.

**Results:**

Post-admission, medication and PIM use increased notably, especially in the first month, with 26% and 34% of residents experiencing increases, respectively. Deprescribing peaked in the second quarter, with 21% of residents reducing their medication use and 25% reducing PIM use. Residents with escalations in medication usage faced a 61% higher risk of hospitalization by the final quarter, while those reducing their medication count had a 21% lower risk by the second quarter. Increases in PIM use were linked to higher hospitalization risks (Quarter 1 (Q1): aHR 1.55 [1.38–1.75], Q4: aHR 1.80 [1.48–2.19]). Conversely, reductions in such use did not significantly alter hospitalization risk.

**Conclusion:**

These findings underscore the need for targeted interventions to manage polypharmacy and PIM use effectively in this population.

## Introduction

The rapid rise in the elderly population and corresponding demand for nursing home (NH) services highlight the widespread issue of polypharmacy and potentially inappropriate medication (PIM) use among NH residents. This concern is particularly acute given the higher rates of emergency department (ED) visits and hospitalizations for NH residents compared to community-dwelling older adults (Dwyer et al., 2014; Trivedi et al., 2018), often leading to significant medical distress and a high burden of care (Kapoor et al., 2019).

While numerous studies have linked polypharmacy and PIM use to increased risk of hospitalization or ED visits (Lalic et al., 2016; Chang et al., 2022; Chae et al., 2023), the majority of these are cross-sectional and do not explore the evolution of PIM usage throughout an individual’s NH residency. Although some studies tracked PIM use trends over time, the findings vary widely due to factors like country, study year, and medication management practices. For instance, some studies report an increase in PIM use over time (Gustafsson et al., 2015; Morin et al., 2016), while others note a decrease (Gustafsson et al., 2015; Cateau et al., 2021). In contrast, a study using the STOPP-Frail (Screening Tool of Older Person’s potentially inappropriate Prescriptions in Frail Older Adults with limited life expectancy) assessment tool noted a decline in PIM use among frail residents over time (Bruin-Huisman et al., 2017), whereas another study observed no change in PIM use among residents with dementia across 2 years (Lalic et al., 2016; Ramsey et al., 2018).

The health and functional status of elderly NH residents often deteriorates following admission (Green et al., 2017). However, there is a notable gap in research regarding how changes in PIM usage, driven by shifts in health status, affect adverse outcomes. Understanding the dynamics of PIM use relative to health status changes is essential to pinpoint high-risk periods and implement targeted interventions.

Deprescribing, defined as the discontinuation of inappropriate medications to minimize adverse drug reactions (Scott et al., 2015), has emerged as a viable strategy to reduce medical transfers (Scott et al., 2015; Wouters et al., 2017; Chang et al., 2023). Although some randomized clinical trials (RCTs) on deprescribing interventions among NH residents have shown promising results in reducing PIM use, their impact on health outcomes is mixed and often inconclusive (Kua et al., 2019). While some studies show no significant difference in hospitalization rates between control and intervention groups (Roberts et al., 2001; Potter et al., 2016), others suggest that medication review–directed deprescribing interventions may reduce all-cause mortality (Kua et al., 2019; Kua et al., 2021). Challenges such as outcome variability and small sample sizes in control groups complicate the comprehensive understanding of deprescribing’s effects. Moreover, these interventions are often undermined by a lack of insufficient understanding of PIM usage patterns.

In South Korea, NHs do not employ in-house physicians. Instead, medical care is provided by contracted physicians who usually visit facilities about twice per week to prescribe medications when needed, while routine daily care is delivered primarily by nurses, nursing assistants, and care workers. In addition, many residents continue to receive prescriptions from their previous outpatient clinics or tertiary hospitals, particularly for chronic diseases. These structural characteristics of Korean NHs may influence patterns of medication use and subsequent health outcomes.

To our knowledge, longitudinal studies that examine changes in PIM use following NH admission and the impact of deprescribing on hospitalization rates are distinctly lacking. This gap in knowledge prompts our current investigation into the evolution of polypharmacy and PIMs, to identify changes in the usage of PIM after admission, and to determine whether these changes correlate with higher hospitalization risks compared to consistent PIM use.

## Methods

### Study population

We utilized senior cohort claims data from the National Health Insurance Service-Senior Cohort (NHIS-SC), covering the period from 2008 to 2019, for a retrospective descriptive analysis. The National Health Insurance Service (NHIS) in South Korea serves as the country’s single public health insurer, providing universal health coverage and also administering Long-Term Care (LTC) insurance. Eligibility for LTC benefits is determined by an LTC grade, which is based on a diagnosis of dementia and an assessment of physical and cognitive function.

This study used data from the NHIS-Senior Cohort, a nationally representative database that includes 18 years (2002–2019) of cumulative records. The cohort was constructed from a stratified random sample of 511,953 older adults who were aged 60 to 80 in 2008, with 8% of newly eligible individuals (aged 60) added each year from 2009 to 2018. All data were de-identified and included demographic characteristics, medical service utilization, and LTC service information such as LTC grade (Chae et al., 2023).

We identified residents aged 65 and older admitted to NHs between July 2008 and December 2018, with their initial first NH stay being at least 30 days. Records were merged for residents readmitted within 7 days after discharge. We excluded residents who received no prescription drugs or who were prescribed only topical preparations during the study period.

This study was approved by the Seoul National University Institutional Review Board (No. E2101/001–001) and the need for informed consent was waived as the study used only de-identified data with no linkable data elements. All methods were adhered to the declaration of Helsinki.

### Assessment of changes in medication and PIM use

Initially, we calculated the total number of medications prescribed for a minimum of 5 days within each assessment period. Based on these data, we defined polypharmacy as the current use of five or more medications and categorized it into 4 levels: 0, low level (1–4), medium level (5–9), and high level (10+). We defined a change in the number of medications as an “addition” when there was an increase to a higher level compared to the previous quarter, and as a “reduction” when there was a decrease to a lower level, with the first quarter’s comparison made against the medication count at the time of admission.

Similarly, the number of PIMs was determined using the 2019 Beers criteria from the American Geriatrics Society. Each component of fixed-dose combination products was evaluated individually. For quarterly assessment, based on the first month of each quarter, we compared the quantity of PIM to the previous quarter. These were categorized as three groups: “addition” for any increase, “maintenance” for no change, and “reduction” for any decrease in components. If the initial count exceeds five, a ‘reduction’ was specifically defined as decreasing to five or fewer. Only individuals who remained alive throughout each assessment period were included in these estimates.

### Association of changes in PIM use with hospitalization

The primary outcome was hospitalization leading to NH discharge. This include hospitalizations on the day of discharge or within 7 days post-discharge, and cases where residents had been hospitalized during their stay in the NH and continued hospitalization after leaving. Death was considered a competing event, occurring either on the day of discharge or within 7 days without any further medical records. Discharges for unspecified reasons were treated as censoring.

**FIGURE 1:**
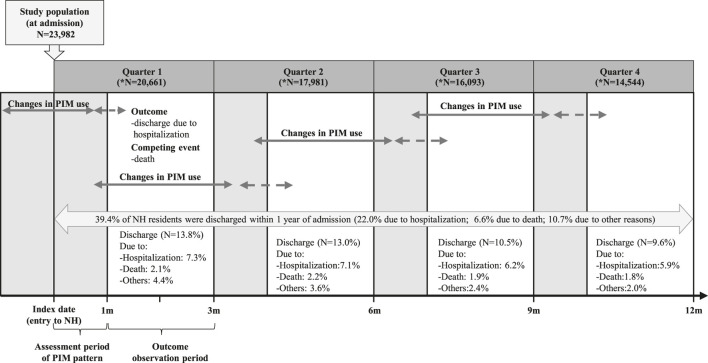
Conceptual framework for analyzing the association between changes in potentially inappropriate medication use and hospitalization after nursing home admission. Note: “N” represents the number of residents remaining at the end of each quarter.

We analyzed medication claims and medical records from the first year of NH admission to assess changes in PIM use during the first month of each quarter and their association with subsequent hospitalizations in the following 2 months (Figure 1).

### Statistical analysis

Demographic information and pre-existing conditions of residents were collected at the time of their admission to the NH, analyzed using descriptive statistics. Pre-existing conditions were identified based on the International Classification of Diseases-10 (ICD-10) codes for the year preceding NH admission. To explore the relationship between changes in PIM use and the risk of hospitalization, we applied a cause-specific hazard model, adjusting for range of potential confounders. These included age, sex, long-term care grade, Charlson comorbidity index (CCI), and specific comorbidities such as dementia, hypertension, diabetes, ischemic heart disease, heart failure, chronic obstructive pulmonary disease, asthma, peptic ulcer disease, history of fall, history of pneumonia, angina and cancer. All statistical analyses were conducted using SAS Enterprise Guide software, version 8.3 (SAS Institute, Inc., Cary, NC) with a 95% CI, and a p-value <0.05 was considered statistically significant.

## Results

### Characteristics of residents

Our study included 23,982 older adults admitted to NHs between July 2008 and December 2018. Over half of the participants were aged 75–84 years, and approximately 70% were female. The most common comorbidities were dementia and hypertension, affecting 73.0% and 71.9% of the residents, respectively (Table 1). Within the first year of admission, 9,438 (39.4%) residents were discharged; 22% were due to hospitalization, and 6.6% were due to mortality. The highest rate of discharge occurred in the first quarter (Figure 1).

**TABLE 1:** Baseline characteristics of the study population (N = 23,982).

Characteristics	N (%)
Sex, female	16,766 (69.9)
Age, years, mean ± SD	78.7 ± 5.7
65–69	1812 (7.6)
70–74	3,752 (15.7)
75–79	6,897 (28.8)
80–84	7,673 (32.0)
≥85	3,848 (16.1)
Insurance type	
Health insurance	19,798 (82.6)
Medical aid	4,184 (17.4)
Disability level	
Non-disabled	15,337 (63.9)
Moderate disability	4,933 (20.6)
Severe disability	3,712 (15.5)
LTC grade	
Grade 1	2,891 (12.1)
Grade 2	6,250 (26.1)
Grade 3 and below	14,841 (61.9)
CCI, mean ± SD	3.5 ± 2.2
0	1,146 (4.8)
1–4	15,888 (66.3)
≥5	6,948 (29.0)
Comorbidities^a^	
Dementia	17,502 (73.0)
Hypertension	17,250 (71.9)
Diabetes	10,103 (42.1)
COPD	6,343 (26.4)
History of fall	5,790 (24.1)
Ischemic heart disease	4,526 (18.9)
History of pneumonia	3,600 (15.0)
Heart failure	3,451 (14.4)
Asthma	3,285 (13.7)
Parkinson’s disease	3,115 (13.0)
Cancer	1,563 (6.5)

^a^ Based on diseases within 1 year prior to nursing home admission.

LTC, long-term care; CCI, Charlson comorbidity index; COPD, chronic obstructive pulmonary disease.

### Changes in polypharmacy and PIM use

After admission, there was a notable increase in medication use, with an average starting at 8.3 ± 6.6 medications per resident. Nearly 70% of residents were initially experiencing polypharmacy (Supplementary Figure S1; Supplementary Table S1). This figure escalated to 81% after 1 month averaging 9.3 ± 5.7 medications per resident, with those taking more than 10 medications rising from 38% to 43%. By the end of the first year, around 80% of residents continued to engage in polypharmacy, marking a 10%p increase from admission.

Similarly, the use of PIMs increased within the first year (Supplementary Figure S1 and Supplementary Table S1). Initially, 74% of residents (averaging 2.4 ± 2.4 PIMs per resident) were taking PIMs in the first month, indicating a 14%p increase from admission. The most substantial increases were observed in residents taking one or two PIMs (35%) followed by those using three to four PIMs (21%), and more than five PIMs (18%). PIM usage then stabilized at 71% after 3 months.

**FIGURE 2:**
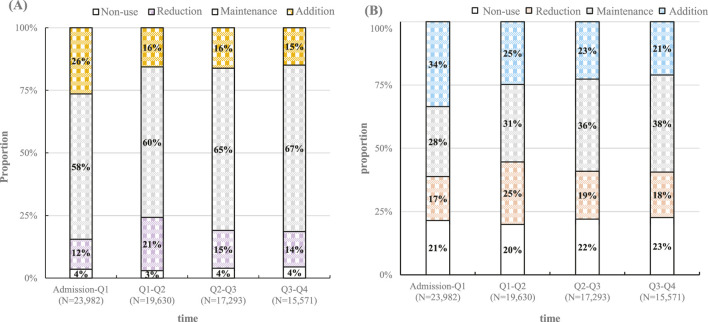
Temporal changes in **(A)** overall medication use and **(B)** potentially inappropriate medication use after nursing home admission. Note: “N” represents the number of residents remaining in the nursing home during the first month of each quarter.

Over half of the residents maintained their level of medication usage over time, with this figure rising to 67% (Figure 2A). In the first quarter post-admission, 26% of residents increased their medication usage, while 21% reduced it by the second quarter. The trends in PIM additions and reductions mirrored this pattern, with 34% of residents increasing their PIM use initially and 25% decreasing by the second quarter. By the last quarter, 38% of residents had maintained their PIM usage level (Figure 2B). Notably, the most significant changes in both medication and PIM usage occurred within the first 6 months, stabilizing thereafter.

### Changes in specific PIM prevalence

First-generation antihistamines, antipsychotics, benzodiazepines, and NSAIDs (when prescribed in high-risk patients based on Beers criteria) were the most frequently used PIMs. Antipsychotics and benzodiazepines showed significant increases in usage during the first quarter by 11%p and 10%p respectively, maintaining high rates thereafter. NSAIDs were commonly used short-term, with a continued usage rate of 7%. The usage of first-generation antihistamines varied, reflecting their wide range of applications (Supplementary Table S2).

### Association of Polypharmacy/PIM use with hospitalization

**FIGURE 3:**
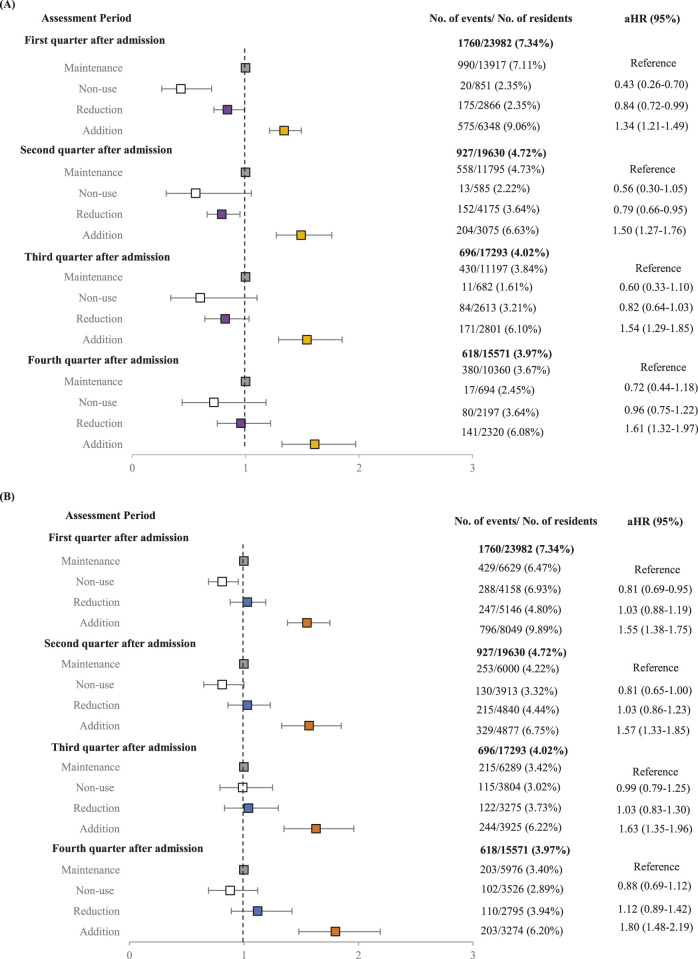
Forest plot of adjusted hazard ratios describing the association of **(A)** overall medication use and **(B)** potentially inappropriate medication use with the risk of hospitalization after nursing home admission. *Adjusted variables were age, sex, long‐term care grade, Charlson comorbidity Index, dementia, hypertension, diabetes, parkinson, chronic obstructive pulmonary disease, peptic ulcer disease, history of fall, ischemic heart disease, heart failure, asthma, history of pneumonia, angina, cancer.

Residents increasing their medication count faced a heightened risk of hospitalization, with a 34% increased risk observed in the 2 months following a medication increase in the first month after admission. Those with sustained escalations in medication usage experienced a 61% higher risk of hospitalization by the final quarter. Conversely, during the first 6 months, those who reduced their medication count saw a beneficial decrease in hospitalization risk compared to those who maintained their medication levels. Notably, in the second quarter, when the most significant medication reduction occurred, the group that reduced their medication count exhibited a 21% lower hospitalization risk (Figure 3A). Similarly, residents with added PIMs faced comparable hospitalization risks. An initial increase in PIM use after admission was associated with a 55% higher risk of hospitalization in the subsequent 2 months. Moreover, those with sustained escalation in PIM use experienced a 1.8-fold increase in hospitalization risk by the final quarter. However, no significant reduction in hospitalization risk was observed among residents who reduced their PIM use during their stay (Figure 3B).

## Discussion

To the best of our knowledge, this is the first study to investigate the complex dynamics of medication and PIM use among NH residents in Korea, offering valuable insights into how these patterns relate to hospitalization risk. This study reveals a notable increase in both medication and PIM usage immediately following NH admission, aligning with findings from Danish and Canadian research that observed similar trends post-admission (Lundby et al., 2020; Maclagan et al., 2017). A particularly striking is the significant fluctuation in medication and PIM counts within the first 6 months of residency, underlining this period as critical for intervention.

The initial surge in PIM usage, most pronounced in the first quarter, coupled with substantial reductions in the subsequent quarter, reflects the dynamic adjustments in medication management during the early months of NH residency. This pattern is consistent with literature that shows a prevalent reliance on antipsychotics and benzodiazepines within NH settings, often as a response to adjustment challenges in new living environments (Rochon et al., 2007; Olsson et al., 2010; Neft et al., 2020). Our observations supported by previous findings (Jang et al., 2022), suggest that the increase in PIM use, particularly benzodiazepines and antipsychotics, may serve short-term treatments to alleviate transitional stress or other medical needs post-admission, including manage conditions like insomnia that may exacerbate during such transitions.

The second quarter post-admission stands out as a key period for observed reductions, particularly in the use of benzodiazepines and antipsychotics, underscoring the importance of adjusting medication use early in residency. Despite frequent prescriptions, NSAIDs were typically used short-term, contrasting with benzodiazepines and antipsychotics. The significant use of first-generation antihistamines reflects their broad applications and affordability (Jang et al., 2022).

In line with previous findings (Allin et al., 2017; Chang et al., 2020; Weir et al., 2020), we observed that increased medication and PIM use are associated with elevated hospitalization risks. However, we observed no significant difference in hospitalization risk between residents who reduced their PIM use and those who remained constant. Despite the questions raised in literature about the effectiveness of targeting medications deemed inappropriate by Screening Tool of Older Person’s Prescriptions (STOPP) criteria and Beers criteria for deprescription (Holmes and Sachs, 2017), our findings did not reveal an increased hospitalization risk from discontinuing PIM, echoing similar studies (Scott et al., 2022). This observation aligns with other RCTs suggesting that deprescribing PIMs does not increase hospitalization risk, highlighting benefits of avoiding unnecessary costs, waste, and reducing pill burden, consistent with prior observations (McDonald et al., 2022).

Moreover, a decrease in the overall number of medications early in the NH stay was linked to a reduce risk of hospitalization, suggesting a potential benefit in early medication review and adjustment upon NH admission.

Our research leverages national claims data from South Korea, for the first time, to uncover patterns of PIM use over time and their correlation with hospitalization risk among NH. Nonetheless, several limitations should be considered.

First, the study relies on claims data, which are primarily generated for billing purposes and do not verify actual medication intake or capture over-the-counter and as-needed medications. In addition, clinical reasons underlying changes in PIM use, such as disease progression or medication adjustments, are not available. This restricts our ability to fully interpret whether observed changes in prescribing patterns reflect patient needs, clinical decision-making, or inappropriate prescribing.

Second, although this observational cohort study adjusted for multiple potential confounders (e.g., age, sex, LTC grade, CCI, and comorbidities), unmeasured confounding could not be ruled out in the absence of randomization. Moreover, important patient-level clinical variables such as lifestyle factors, functional status, nutritional status, and mental health status, which may substantially influence the risk of hospitalization, were not captured in claims data, further raising the possibility of residual confounding. Therefore, while we observed an association between PIM burden and hospitalization risk, causality cannot be established. Furthermore, although hospitalization diagnoses (e.g., ICD-10 codes) are available in the dataset, the lack of accompanying clinical information limits our ability to determine whether hospitalizations were directly triggered by medication-related issues.

Third, this study included only residents with at least one prescription during the observation period, potentially excluding healthier individuals without medication use and introducing selection bias. Nevertheless, the nationwide claims cohort ensures reasonable representativeness. In addition, residents’ health status may deteriorate over time, which could simultaneously increase both medication burden and hospitalization risk, contributing to time-related bias. To reduce the impact of attrition due to death or loss to follow-up, we restricted the primary risk analysis to a fixed 3-month intervals.

Fourth, the wide dispersion of residents and the merging of hospitalization records across facilities limited our ability to evaluate whether NH characteristics influenced medication use or hospitalization risk.

Finally, as an observational study, our analysis cannot provide the same level of causal inference as randomized controlled trials. Future high-quality prospective studies, including RCTs or target trial emulation studies, are warranted to validate whether targeted interventions to reduce PIMs can meaningfully improve clinical outcomes.

In conclusion, this study observed a temporal increase in PIM use after NH admission, which was associated with higher risk of hospitalization. However, no significant association was found between reductions in PIM use and lower risk, possibly reflecting the complexity of medication use in NH settings. These findings suggest the need for further investigation into optimal medication management strategies, particularly during the early post-admission phase.

## Data Availability

The datasets presented in this article are not readily available because The dataset that supports the findings of this article can be obtained from the Korea National Health Insurance Service (KNHIS) Data Sharing Service homepage (https://nhiss.nhis.or.kr/). However, access to the data is restricted. The data provider, KNHIS, mandates that all researchers pledge not to share, release, or review the data with any external parties. Requests to access the datasets should be directed to https://nhiss.nhis.or.kr/.
