# Supplementary material for: Impact of post-admission changes in potentially inappropriate medication use on risk of subsequent hospitalization among nursing home residents
Source: Front Pharmacol. 2025 Sep 23;16:1655681. doi: 10.3389/fphar.2025.1655681 (PMC12500243; doi:10.3389/fphar.2025.1655681)
Supplement: Supplementary file 1 [file DataSheet1.docx]

**Supplementary material**

**Title: Impact of post-admission changes in potentially inappropriate medication use on risk of subsequent hospitalization among nursing home residents**

- Supplementary Figure S1. Trends in medications and potentially inappropriate medications use after admission to nursing home by quarter
- Supplementary Table S1. The average number of medications and potentially inappropriate medications used per person after admission to the nursing home
- Supplementary Table S2. Usage patterns of specific potentially inappropriate medications after nursing home admission.

**Supplementary Figure S1. Trends in (A) overall medication use and (B) potentially inappropriate medication use after nursing home admission.** Note: The prevalence refers to residents who remained in the nursing home during the first month of each quarter. (Admission: N=23,982; Q1: N=23,982; Q2: N=19,630; Q3: N=17,293; Q4: N=15,571)

| **Supplementary Table S1.** **The average number of medications and potentially inappropriate medications used per person after admission to the nursing home** | | | | | |
| --- | --- | --- | --- | --- | --- |
| Period | Admission  *N=23,982 | Q1  *N=23,982 | Q2  *N=19,630 | Q3  *N=17,293 | Q4  *N=15,571 |
| No. of medication | 8.34±6.61 | 9.30±5.68 | 8.42±5.20 | 8.30±5.03 | 8.28±5.08 |
| No. of PIMs | 2.04±2.59 | 2.39±2.43 | 2.23±2.30 | 2.21±2.25 | 2.20±2.25 |
| Note: "N" represents the number of residents remaining in the nursing home in the first month of each quarter. | | | | | |

| **Supplementary Table S2. Usage patterns of specific potentially inappropriate medications after nursing home admission.** | | | | |
| --- | --- | --- | --- | --- |
| **PIM component** | **Admission-Q1**  **(*N=23,982)** | **Q1-Q2**  **(*N=19,630)** | **Q2-Q3**  **(*N=17,293)** | **Q3-Q4**  **(*N=15,571)** |
| **Antipsychotics** | | | | |
| Reduction | 881 (3.7) | 1,190 (6.1) | 704 (4.1) | 563 (3.6) |
| Maintenance | 3,726 (15.5) | 3,955 (20.2) | 4,097 (23.7) | 3,928 (25.2) |
| Addition | 2,695 (11.2) | 1,354 (3.9) | 892 (5.2) | 654 (4.2) |
| **Benzodiazepines** | | | | |
| Reduction | 1,308 (5.5) | 1,571 (8.0) | 956 (5.5) | 690 (4.4) |
| Maintenance | 3,528 (14.7) | 3,246 (16.5) | 3,167 (18.3) | 3,017 (19.4) |
| Addition | 2,444 (10.2) | 1,277 (6.5) | 885 (5.1) | 690 (4.4) |
| **NSAIDs*** | | | | |
| Reduction | 1,986 (8.3) | 1,806 (9.2) | 1,122 (6.5) | 978 (6.3) |
| Maintenance | 1,457 (6.1) | 1,210 (6.2) | 1,226 (7.1) | 1,126 (7.2) |
| Addition | 2,066 (8.6) | 1,411 (7.2) | 1,162 (6.7) | 942 (6.1) |
| **First-generation antihistamines** | | | | |
| Reduction | 1,430 (6.0) | 1,611 (8.2) | 1,299 (7.5) | 1,184 (7.6) |
| Maintenance | 1,281 (5.3) | 957 (4.9) | 951 (5.5) | 928 (6.0) |
| Addition | 2,045 (8.5) | 1,727 (8.8) | 1,432 (8.3) | 1,310 (8.4) |
| **Sulfonylureas** | | | | |
| Reduction | 235 (1.0) | 354 (1.8) | 182 (1.1) | 127 (0.8) |
| Maintenance | 1,889 (7.9) | 1,661 (8.5) | 1,403 (8.1) | 1,243 (8.0) |
| Addition | 560 (2.3) | 155 (0.8) | 108 (0.6) | 81 (0.5) |
| **Nonbenzodiazepine, benzodiazepine receptor agonist hypnotics (“Z-drugs”)** | | | | |
| Reduction | 415 (1.7) | 619 (3.2) | 380 (2.2) | 236 (1.5) |
| Maintenance | 1,199 (5.0) | 1,221 (6.2) | 1,168 (6.8) | 1,107 (7.1) |
| Addition | 1,093 (4.6) | 567 (2.9) | 342 (2.0) | 240 (1.5) |
| **Proton-pump inhibitors** | | | | |
| Reduction | 693 (2.9) | 619 (3.2) | 337 (2.0) | 293 (1.9) |
| Maintenance | 1,300 (5.4) | 1,040 (5.3) | 1,010 (5.8) | 953 (6.1) |
| Addition | 789 (3.3) | 491 (2.5) | 393 (2.3) | 314 (2.0) |
| **Antidepressants** | | | | |
| Reduction | 266 (1.1) | 337 (1.7) | 213 (1.2) | 145 (0.9) |
| Maintenance | 1083 (4.5) | 997 (5.1) | 904 (5.2) | 840 (5.4) |
| Addition | 547 (2.3) | 269 (1.4) | 186 (1.1) | 152 (1.0) |
| **Digoxin** | | | | |
| Reduction | 51 (0.2) | 63 (0.3) | 27 (0.2) | 23 (0.2) |
| Maintenance | 544 (2.3) | 442 (2.3) | 378 (2.2) | 336 (2.2) |
| Addition | 140 (0.6) | 45 (0.2) | 27 (0.2) | 26 (0.2) |
| **Antiparkinsonian agents** | | | | |
| Reduction | 177 (0.7) | 181 (0.9) | 99 (0.6) | 73 (0.5) |
| Maintenance | 565 (2.4) | 468 (2.4) | 460 (2.7) | 440 (2.8) |
| Addition | 228 (1.0) | 160 (0.8) | 116 (0.7) | 87 (0.6) |
| **Insulin** | | | | |
| Reduction | 309 (1.3) | 807 (4.1) | 199 (1.2) | 135 (0.9) |
| Maintenance | 1263 (5.3) | 264 (1.3) | 194 (1.1) | 155 (1.0) |
| Addition | 205 (0.9) | 190 (1.0) | 160 (0.9) | 130 (0.8) |
| **Metoclopramide** | | | | |
| Reduction | 275 (1.2) | 169 (0.9) | 98 (0.6) | 74 (0.5) |
| Maintenance | 161 (0.7) | 110 (0.6) | 82 (0.5) | 81 (0.5) |
| Addition | 237 (1.0) | 126 (0.6) | 106 (0.6) | 85 (0.6) |
| **Peripheral alpha-1 blockers** | | | | |
| Reduction | 31 (0.1) | 85 (0.4) | 30 (0.2) | 19 (0.1) |
| Maintenance | 142 (0.6) | 129 (0.7) | 113 (0.7) | 115 (0.7) |
| Addition | 125 (0.5) | 39 (0.2) | 33 (0.2) | 18 (0.1) |
| **Antispasmodics** | | | | |
| Reduction | 362 (1.5) | 176 (0.9) | 104 (0.6) | 83 (0.5) |
| Maintenance | 95 (0.4) | 61 (0.3) | 55 (0.3) | 55 (0.4) |
| Addition | 223 (0.9) | 137 (0.7) | 114 (0.7) | 80 (0.5) |
| **Amiodarone** | | | | |
| Reduction | 12 (0.1) | 10 (0.1) | 3 (0.0) | 6 (0.0) |
| Maintenance | 59 (0.3) | 46 (0.2) | 37 (0.2) | 30 (0.2) |
| Addition | 15 (0.1) | 8 (0.0) | 8 (0.1) | 4 (0.0) |
| **Skeletal muscle relaxants** | | | | |
| Reduction | 140 (0.6) | 83 (0.4) | 54 (0.3) | 36 (0.2) |
| Maintenance | 70 (0.3) | 42 (0.2) | 45 (0.3) | 40 (0.3) |
| Addition | 88 (0.4) | 68 (0.4) | 37 (0.2) | 34 (0.2) |
| **Desmopressin** | | | | |
| Reduction | 29 (0.1) | 21 (0.1) | 11 (0.1) | 5 (0.0) |
| Maintenance | 63 (0.3) | 38 (0.2) | 34 (0.2) | 26 (0.2) |
| Addition | 13 (0.1) | 11 (0.1) | 5 (0.0) | 6 (0.0) |
| **Megestrol** | | | | |
| Reduction | 41 (0.2) | 34 (0.2) | 21 (0.2) | 11 (0.1) |
| Maintenance | 64 (0.3) | 24 (0.1) | 13 (0.1) | 8 (0.1) |
| Addition | 52 (0.2) | 28 (0.1) | 17 (0.1) | 14 (0.1) |
| **Nifedipine** | | | | |
| Reduction | 5 (0.0) | 14 (0.1) | 8 (0.1) | 2 (0.0) |
| Maintenance | 32 (0.1) | 17 (0.1) | 14 (0.1) | 14 (0.1) |
| Addition | 10 (0.0) | 6 (0.0) | 2 (0.0) | 1 (0.0) |
| **Barbiturates** | | | | |
| Reduction | 9 (0.0) | 7 (0.0) | 8 (0.0) | 4 (0.0) |
| Maintenance | 16 (0.1) | 16 (0.1) | 16 (0.1) | 13 (0.1) |
| Addition | 12 (0.1) | 8 (0.0) | 5 (0.0) | 1 (0.0) |
| **Dipyridamole** | | | | |
| Reduction | 0 (0.0) | 0 (0.0) | 2 (0.0) | 0 (0.0) |
| Maintenance | 6 (0.0) | 4 (0.0) | 3 (0.0) | 5 (0.0) |
| Addition | 1 (0.0) | 2 (0.0) | 2 (0.0) | 0 (0.0) |
| **Estrogens with or without progestins** | | | | |
| Reduction | 1 (0.0) | 6 (0.0) | 1 (0.0) | 2 (0.0) |
| Maintenance | 6 (0.0) | 2 (0.0) | 2 (0.0) | 2 (0.0) |
| Addition | 3 (0.0) | 1 (0.0) | 3 (0.0) | 1 (0.0) |
| **Ketorolac** | | | | |
| Reduction | 8 (0.0) | 0 (0.0) | 0 (0.0) | 2 (0.0) |
| Maintenance | 0 (0.0) | 0 (0.0) | 0 (0.0) | 0 (0.0) |
| Addition | 1 (0.0) | 0 (0.0) | 2 (0.0) | 1 (0.0) |
| **Ergoloid mesylates** |  |  |  |  |
| Reduction | 0 (0.0) | 0 (0.0) | 0 (0.0) | 0 (0.0) |
| Maintenance | 0 (0.0) | 0 (0.0) | 0 (0.0) | 0 (0.0) |
| Addition | 0 (0.0) | 0 (0.0) | 0 (0.0) | 0 (0.0) |
| *NSAIDs (Non-Steroidal Anti-Inflammatory Drugs): residents in high-risk groups, including those >75 years or taking oral or parenteral corticosteroids, anticoagulants, or antiplatelet agents.  Note: "N" represents the number of residents remaining in the nursing home in the first month of each quarter. | | | | |
